# Work/household, transport, and leisure domains account for the sex gap in physical activity in Chile

**DOI:** 10.3389/fpubh.2022.1011790

**Published:** 2022-09-28

**Authors:** Mónica Suárez-Reyes, Rodrigo Fernández-Verdejo

**Affiliations:** ^1^Escuela de Ciencias de la Actividad Física, el Deporte y la Salud (ECIADES), Universidad de Santiago de Chile, Santiago, Chile; ^2^Laboratorio de Fisiología del Ejercicio y Metabolismo (LABFEM), Escuela de Kinesiología, Facultad de Medicina, Universidad Finis Terrae, Santiago, Chile

**Keywords:** exercise, health survey, risk factors, health promotion, behavior

## Abstract

**Background:**

Women usually have lower levels of moderate-vigorous physical activity (MVPA) than men. This sex gap can be accounted for by differences in MVPA in the work/household, transport, and/or leisure domains. Identifying where the differences lay in a context-specific manner may help close the gap. We aimed to compare MVPA by domain, and the relative contribution of each domain to total MVPA, between men and women in Chile.

**Methods:**

We analyzed the cross-sectional National Health Survey of Chile 2016–2017 (*n* = 5,056, 64% women, ≥18 years old). MVPA was estimated with the Global Physical Activity Questionnaire. MVPA was expressed in MET × min/week, and the relative contribution to total MVPA by each domain was expressed as percentage. Analyses were conducted including all participants, and also including participants reporting >0 MET × min/week of MVPA (relative contributions can only be computed in the latter).

**Results:**

Including all participants, women (vs. men) had lower MVPA (median [25–75th percentile]) for work/household (0 [0–960] vs. 0 [0–5,760] MET × min/week), for transport (360 [0–1,200] vs. 600 [0–1,680] MET × min/week), and for leisure domains (0 [0–0] vs. 0 [0–480] MET × min/week). Including only participants with >0 MET × min/week of MVPA, women (vs. men) had lower mean relative contributions to total MVPA from work/household (31.3 vs. 35.9%) and leisure domains (10.8 vs. 16.3%, respectively), but higher from the transport domain (57.9 vs. 47.8%).

**Conclusion:**

In Chile, differences in all physical activity domains account for the sex gap in MVPA. Strategies to break job stereotypes, increase opportunities for leisure, and ease active transport are required to encourage MVPA in women.

## Introduction

Regular physical activity helps prevent and treat several chronic diseases (1–4). The World Health Organization (WHO) recommends doing at least 150–300 min of moderate-intensity or 75–150 min of vigorous-intensity physical activity per week, or an equivalent combination of moderate-vigorous physical activity (MVPA) (2). People who do not meet these recommendations are considered physically inactive or insufficiently active (5–7). In 2016, the worldwide prevalence of physical inactivity was 27.5%, with a higher prevalence in women than men (31.7 and 23.4%, respectively) (7). This sex gap in MVPA is a worldwide trend that reaches more than ten percentage points in some regions (7, 8). Women thus seem less protected against chronic diseases.

MVPA can be undertaken at work/household, for transport, and for leisure (2). These represent the physical activity domains. Differences in domain-specific MVPA may explain the sex gap in MVPA. Lower leisure MVPA in women than men has been suggested to explain the gap (7). Evidence supporting this idea comes from analyses of the relative contribution to total MVPA by domains. Large epidemiological studies have shown that work/household and transport domains are responsible for most MVPA (~50 and ~41%, respectively), with the leisure domain having a smaller contribution (~9%) (9–11). And notably, the relative contribution from the leisure domain was shown to be lower in women than men (9, 10). Promoting leisure MVPA in women thus appears one option to close the sex gap in MVPA.

Yet to fully understand the differences between men and women, the absolute values of MVPA by domain should also be considered. Women may have lower MVPA in all domains than men while maintaining similar relative contributions. Moreover, to compute relative contributions, individuals with no MVPA must be excluded as there is no MVPA to distribute between domains. This probably results in a larger proportion of women being excluded compared to men (12). Recently, Strain et al. (10) analyzed the absolute values of MVPA by domain, and the relative contribution to total MVPA by domain in adults (25–64 years old) from 104 countries. MVPA was estimated using the Global Physical Activity Questionnaire (GPAQ), which registers weekly time spent on MVPA by domain (13). In absolute values, women had lower work/household, transport, and leisure MVPA than men. As for relative contributions, women tended to have lower leisure and work/household MVPA, and higher transport MVPA than men. These global data suggest that the sex gap in MVPA comes from all domains. Nevertheless, the large inter-country variability in these trends highlights the need to conduct country-specific analyses to better understand each context. Moreover, whether sex differences still appear among people who meet the physical activity recommendations is unknown. Identifying the domains that contribute to the sex gap, and those more feasible to be intervened in specific contexts may guide future public health policies.

Herein, we focused on the adult population living in Chile. The aims of the study were: [a] to compare MVPA by domain between men and women; and [b] to compare the relative contribution of each domain to total MVPA between men and women.

## Methods

### Design and setting

This was an observational, analytical, cross-sectional study. The reporting methodology followed the STROBE guidelines (Supplementary Table 1). We used data from the Surveys of Health for epidemiologic surveillance by the Public Health Subsecretary of Chile, but our findings do not compromise such Institution. The Scientific Ethics Committee of the Faculty of Medicine of Pontificia Universidad Católica de Chile approved the protocols and written informed consent for the National Health Survey of Chile 2016–2017 (CEC-MedUC, project number 16–019).

In this report, we analyzed the data from the National Health Survey of Chile 2016–2017. This was a cross-sectional household survey that collected data from 6,233 participants (≥15 years old) between August 2016 and March 2017. The methodological details have been described elsewhere (14). The sampling method considered 30 strata (urban and rural areas of 15 geographical regions), and was multistage. The primary sampling units were the counties, followed by households, and finally one participant per household. Herein, we analyzed unweighted variables, because our focus was on comparing MVPA between sexes, not on establishing a national prevalence. We previously reported the national prevalence of physically inactive people in Chile by considering the sampling weights of the survey (6).

### Participants

We included participants who met the following eligibility criteria: [a] were ≥18 years old; [b] had the body mass index (BMI) measured as part of the survey; and [c] had valid physical activity data (see section *MVPA by domain*). Thus, all the participants had basic sociodemographic data (sex, age, and BMI) along with the estimation of physical activity by domain.

### Sociodemographic data

Trained nurses collected the data, and measured weight and height. The details have been described elsewhere (14). BMI was calculated as the quotient between weight (kg) and squared height (m^2^), and participants were then categorized as: underweight (BMI < 18.5 kg/m^2^), normal weight (BMI between 18.5 and <25 kg/m^2^), overweight (BMI between 25.0 and <30.0 kg/m^2^), or obesity (BMI ≥ 30 kg/m^2^). Education was self-reported, and categorized as: <8, 8–12, or >12 years. Marital status was self-reported and categorized as: married or in couple, divorced or separated, widowed, or single. Finally, participants self-reported their occupation status, and this information was categorized as: participants who were either working, studying or searching for work, or participants who were not doing so (e.g. retired). Participants with missing values in one of these variables were excluded from the analyses encompassing such a variable.

### MVPA by domain

MVPA was estimated by the GPAQ (13). Therein, participants report the weekly frequency, and daily time (in at least 10-min bouts) they engaged in activities of moderate and vigorous intensity. This information is obtained for the work/household and leisure domains. Participants also report the weekly frequency, and daily time (in at least 10-min bouts) they engaged in active transport, i.e. walking or cycling (transport domain). Moderate-intensity activities and transport are assumed to require 4 metabolic equivalents (MET), whereas vigorous-intensity activities are assumed to require 8 MET. The level of MVPA by domain (as a continuous variable in MET × min/week) was computed as: weekly frequency (in day/week) × daily time (in min/day) × intensity (in MET) (5, 15). As done before (6), participants were considered to have invalid data if they reported: [a] inconsistent responses, e.g. reporting that they did not engage in vigorous work/household activity, but reporting a daily time in such activity; [b] <10 min of activity in any sub-domain, e.g. vigorous work/household activity; [c] more than 16 h/day in any sub-domain; or [d] more than 10,080 min/week of total activity, including sedentary behavior (which is also included in the GPAQ). Based on the sum of MVPA in all domains, participants were categorized as: no MVPA (0 MET × min/week), insufficiently active (>0 to <600 MET × min/week), or active (≥600 MET × min/week) (5, 15). In participants who reported some MVPA (i.e. insufficiently active, and active groups), we also calculated the relative contribution (%) to total MVPA by domain.

### Statistics

All continuous variables were non-normally distributed according to the Kolmogorov-Smirnov test. Therefore, data for continuous variables are presented as median [25–75th percentile]. Since MVPA data were highly skewed to low values (approaching zero), we plotted the data as histograms of relative frequencies accompanied by the mean and median [25–75th percentile]. Supplementary Figure 1 shows an example of how to read the histograms. Also, the mean values of relative contributions to total MVPA by domain were summarized using ternary plots. Supplementary Figure 2 shows an example of how to read the ternary plots. The independent-samples Mann-Whitney U test was used to compare the distribution of continuous variables between men and women. Data for categorical variables are presented as percentages. The Pearson Chi-square test was used to determine the association between categorical variables and sex. Three sub-analyses were conducted to compare MVPA by domain between men and women, including: [a] all selected participants; [b] only participants who reported some MVPA (i.e. categorized as insufficiently active, and active); and [c] only participants categorized as active. All these analyses were also repeated stratified by age: 18 to <25 years old, 25 to <45 years old, 45 to <65 years old, and ≥65 years old. IBM^®^ SPSS Statistics version 26 was used for analyses, considering a *P*-value lower than 0.05 as statistically significant.

## Results

### General characteristics of participants

Of the 6,233 eligible individuals, 238 were excluded because of age, followed by 726 because of missing BMI data, and finally 213 because of invalid MVPA data. Thus, the final sample included 5,056 participants (1,812 men, and 3,244 women). Table 1 shows their general characteristics. Age and occupational status were not different between men and women. But women had higher BMI, higher prevalence of obesity, lower prevalence of 8 or more years of education, and higher prevalence of widowhood.

**Table 1:** General characteristics of participants.

	**Men**	**Women**	***P*** **-value**
*n* (%)	1,812 (35.8)	3,244 (64.2)	-
Age (years)	51 [34–65]	51 [36–65]	0.219
Weight (kg)	79.1 [69.9–88.3]	69.1 [60.7–79.1]	<0.001
Height (m)	1.68 [1.63–1.73]	1.55 [1.50–1.60]	<0.001
Body mass index (kg/m^2^)	27.9 [25.1–31.0]	28.8 [25.4–32.8]	<0.001
**Body mass index category**			
Underweight (%)	0.8	0.7	< 0.001
Normal weight (%)	23.6	21.5	-
Overweight (%)	43.7	35.7	-
Obesity (%)	32.0	42.0	-
**Education** ^A^			
<8 years	22.0	26.7	<0.001
8–12 years	52.3	51.9	-
>12 years	25.7	21.4	-
**Marital status** ^B^			
Married or in couple (%)	55.9	45.2	<0.001
Divorced or separated (%)	8.5	12.5	-
Widowed (%)	5.6	14.3	-
Single (%)	29.9	28.0	-
Working, studying or searching for work (%)^C^	76.7	76.4	0.805

Values are median (25–75th percentile), or frequencies.

A 16 missing values for men and 29 for women;

B 4 missing values for men and 9 for women;

C 21 missing values for men and 48 for women.

### MVPA by domain including all selected participants

**Figure 1:**
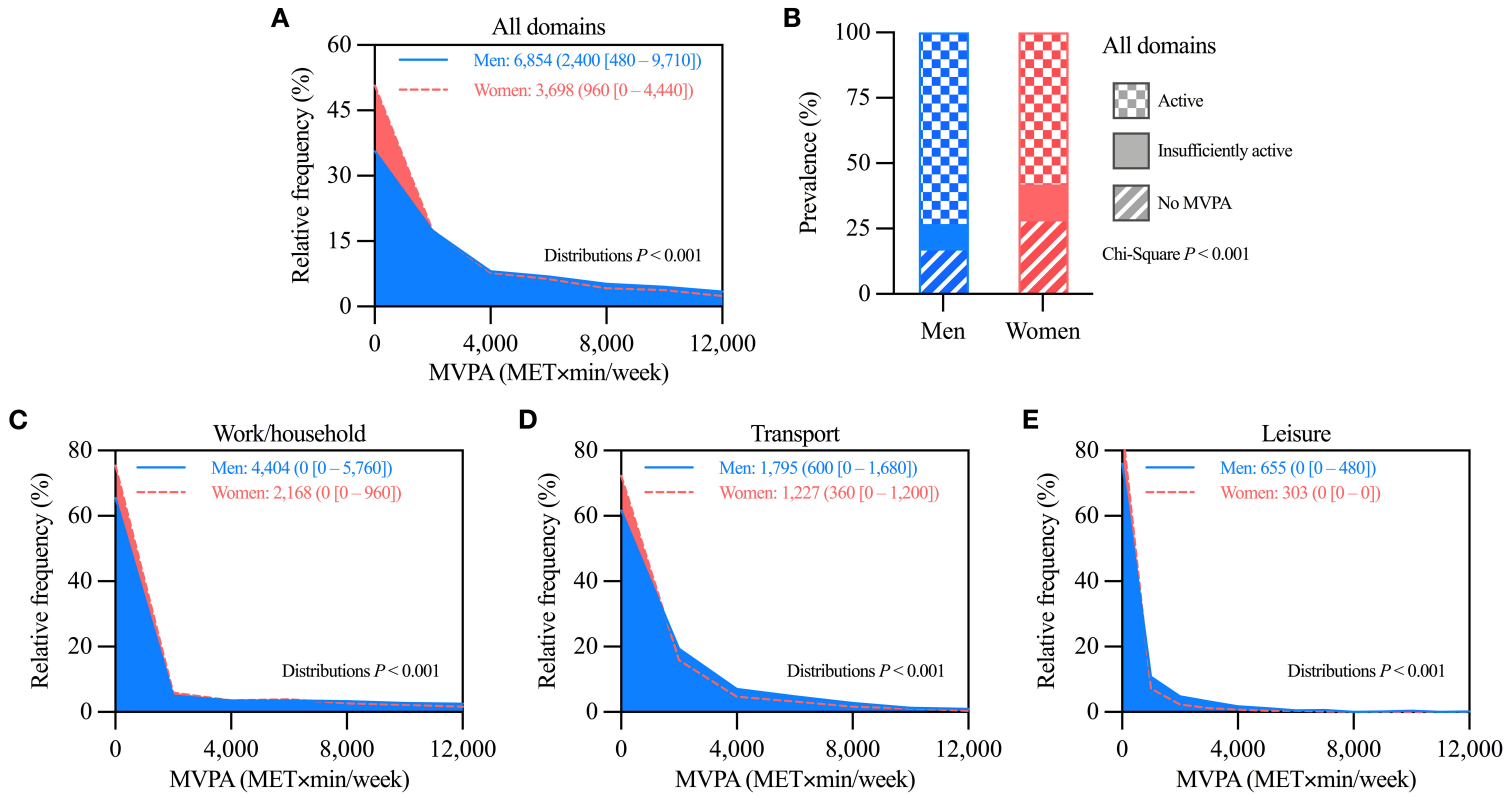
Comparison of moderate-vigorous physical activity (MVPA) levels between all selected men and women. **(A)** Histograms comparing the distributions of MVPA considering all domains. **(B)** Prevalence of MVPA categories. **(C–E)** Histograms comparing the distributions of MVPA in the **(C)** work/household, **(D)** transport, and **(E)** leisure domains. In panels **A** and **C–E**, values are mean (median [25–75th percentile]) of MVPA; distributions more skewed to low MVPA values denote lower MVPA levels. *n* = 1,812 for men, and 3,244 for women.

Considering all domains, MVPA was lower in women than men (Figure 1A). The prevalence of active participants was lower in women than men (58.1 vs. 73.3%, respectively), whereas the prevalence of participants with no MVPA was higher in women than men (28.6 vs. 17.5%, respectively; Figure 1B). Also, women had lower MVPA than men in all domains (Figures 1C–E). Similar trends were observed in the age-stratified analyses (Supplementary Table 2). The only exception was in the ≥65-year-old participants. Therein, men and women had similar MVPA for work/household and for leisure domains (Supplementary Table 2).

### MVPA by domain including only participants with some MVPA

**Figure 2:**
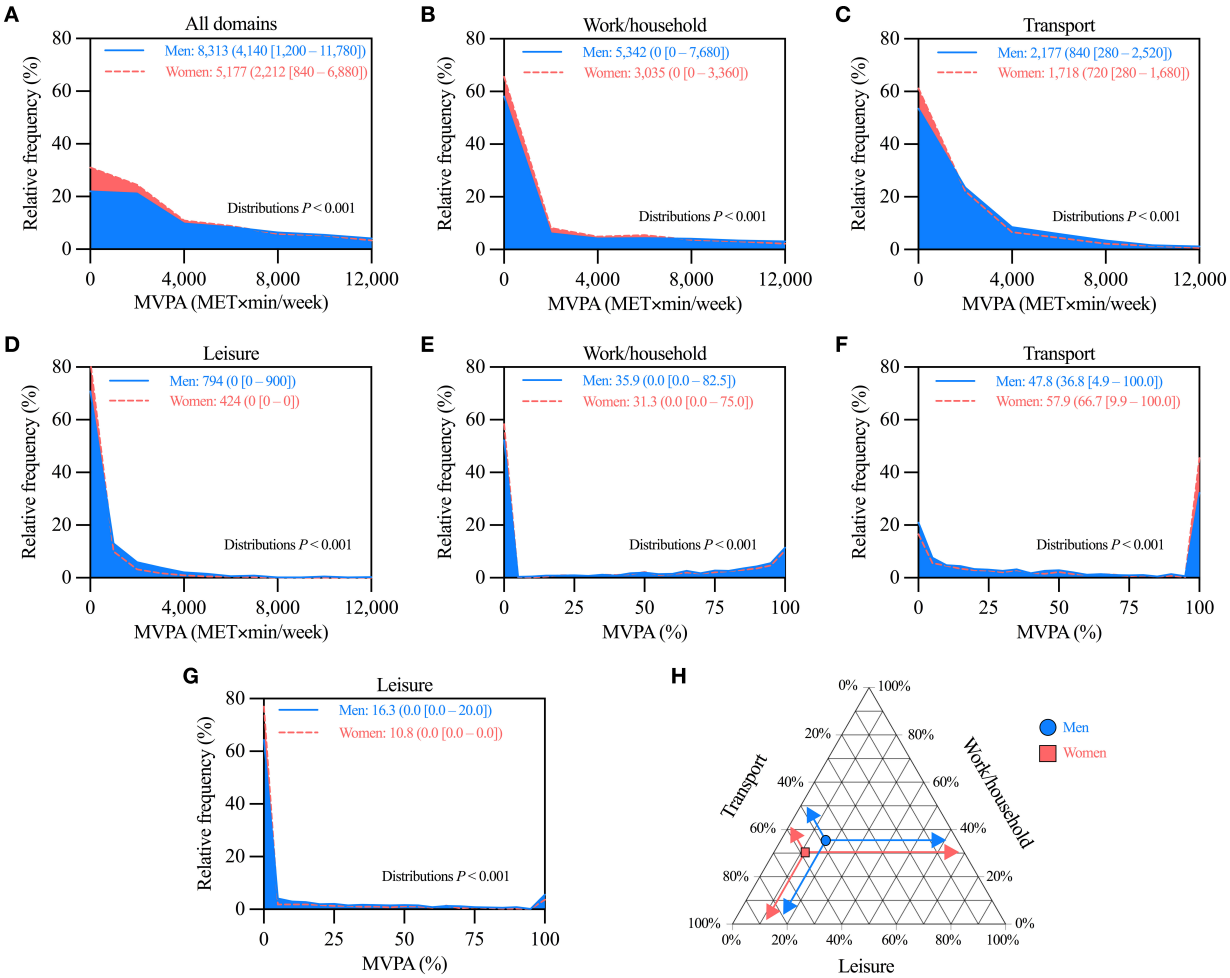
Comparison of moderate-vigorous physical activity (MVPA) levels between men and women that reported some MVPA**. (A–D)** Histograms comparing the distributions of MVPA considering **(A)** all, **(B)** work/household, **(C)** transport, and **(D)** leisure domains. **(E–G)** Histograms comparing the distributions of the relative contribution to MVPA of **(E)** work/household, **(F)** transport, and **(G)** leisure domains. **(H)** Ternary plot summarizing the mean relative contribution to MVPA by domain in men and women. In panels **A–G**, values are mean (median [25–75th percentile]) of MVPA; distributions more skewed to low MVPA values denote lower MVPA. *n* = 1,494 for men, and 2,317 for women.

These analyses included the 3,811 participants (1,494 men, and 2,317 women) that reported some MVPA, i.e. those categorized as insufficiently active or active in Figure 1B. Women had lower MVPA than men when considering all domains, and also in each domain separately (Figures 2A–D). Age-stratified analyses showed similar trends, although with less marked differences, especially among ≥65-year-old participants (Supplementary Table 3). Therein, men and women had similar MVPA for work/household and for leisure domains (Supplementary Table 3).

As for the relative contribution by domain, women (vs. men) had a lower percentage of their MVPA in the work/household and leisure domains, and a higher percentage in the transport domain (Figures 2E–G). Figure 2H summarizes the mean values of relative contributions by domain using a ternary plot. Similar trends were observed in the age-stratified analyses. The only exception was in ≥65-year-old participants. Therein, there were no differences between men and women (Supplementary Table 3).

### MVPA by domain including only participants categorized as active

**Figure 3:**
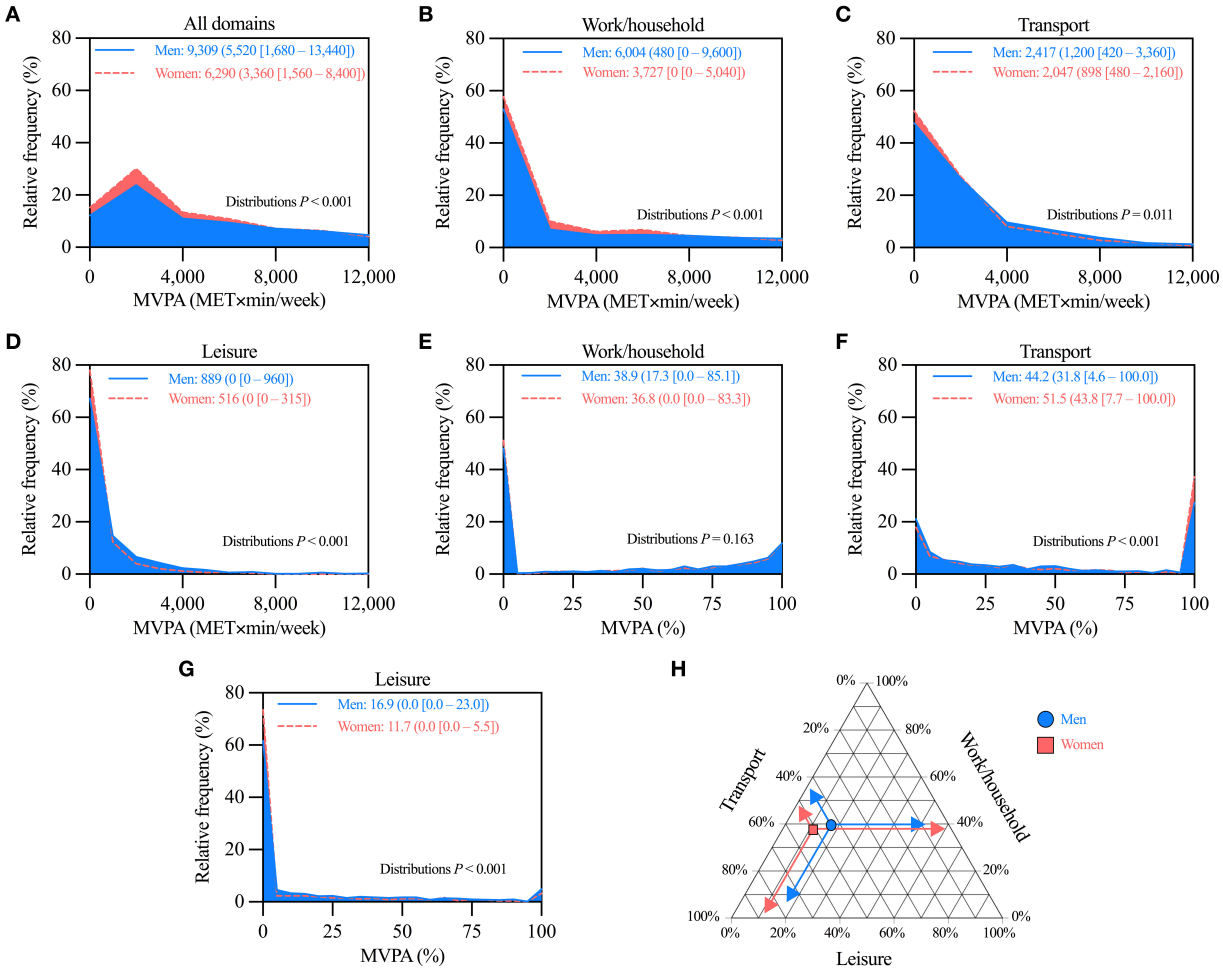
Comparison of moderate-vigorous physical activity (MVPA) levels between men and women categorized as active. **(A–D)** Histograms comparing the distributions of MVPA considering **(A)** all, **(B)** work/household, **(C)** transport, and **(D)** leisure domains. **(E–G)** Histograms comparing the distributions of the relative contribution to MVPA of **(E)** work/household, **(F)** transport, and **(G)** leisure domains. **(H)** Ternary plot summarizing the mean relative contribution to MVPA by domain in men and women. In panels **A–G**, values are mean (median [25–75th percentile]) of MVPA; distributions more skewed to low MVPA values denote lower MVPA. *n* = 1,328 for men, and 1,884 for women.

These analyses included the 3,212 participants (1,328 men, and 1,884 women) that reported ≥600 MET × min/week of MVPA, i.e. those categorized as active in Figure 1B. Women had lower MVPA than men when considering all domains, and also in each domain separately (Figures 3A–D). Similar trends were observed in the age-stratified analyses. The only exception was in the ≥65-year-old participants. Therein, there were no differences between men and women (Supplementary Table 4).

As for the relative contribution by domain, women (vs. men) had a lower percentage of their MVPA in the leisure domain, and a higher percentage in the transport domain. No differences were detected in the work/household domain (Figures 3E–G). Figure 3H summarizes the mean values of relative contributions by domain using a ternary plot. Similar trends were observed in the age-stratified analyses. The only exception was in the ≥65-year-old participants. Therein, there were no differences between men and women (Supplementary Table 4).

## Discussion

**Figure 4:**
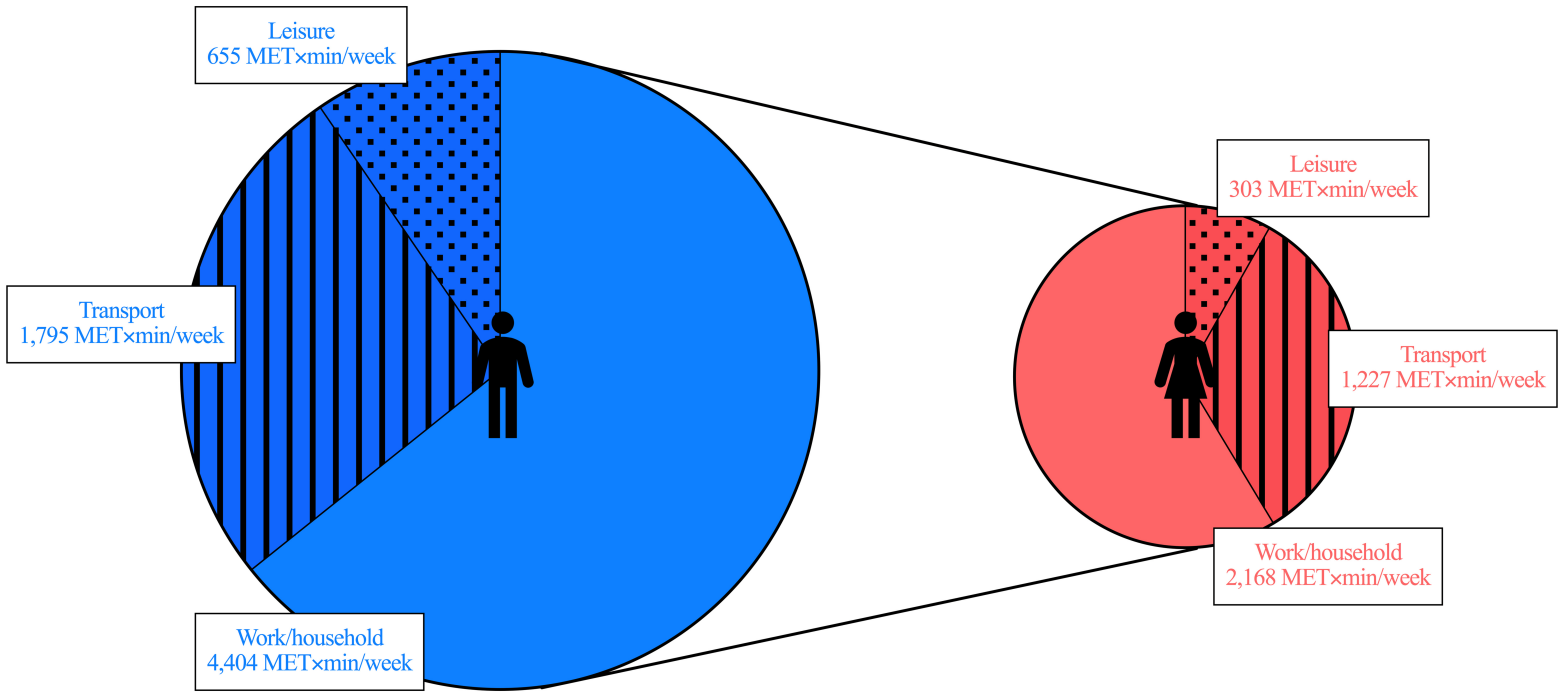
Sex gap in moderate-vigorous physical activity (MVPA) in Chile. Data represent the mean values of MVPA by domain for men **(left)** and women **(right)**. This is a schematic representation of the data from Figures 1C–E.

Worldwide, women are reported to have lower MVPA than men (7, 10). This sex gap may be accounted for by differences in the MVPA conducted for work/household, for transport, and/or for leisure domains (i.e. the physical activity domains). Identifying where the differences lay in a context-specific manner can help design strategies to close the gap. Herein, we compared MVPA by domain, and the relative contribution to total MVPA by domain, between adult men and women in Chile. We found that women had lower MVPA than men in all domains. Notably, the difference appeared when considering all participants—wherein a larger proportion of women had no MVPA–, but also when considering only participants with some MVPA, or only active participants. The relative contribution to total MVPA by domain was also different between sexes. In women, transport represented a larger proportion and leisure a lower proportion of MVPA. Nevertheless, since MVPA consistently differed between sexes, differences in relative contributions by domain could not account for the sex gap. Indeed, most MVPA occurred at work/household in both sexes. The work/household domain thus accounted for most of the differences in MVPA. Figure 4 summarizes the sex gap in terms of the mean MVPA by domain. Strategies promoting MVPA in all domains in women are required to help close the sex gap in Chile.

Previous data from all over the world—including Chile—show that women self-report lower MVPA than men (6, 7, 9, 10). Our current analyses support those observations by showing lower total MVPA in women than men. The mean difference was 3,156 MET × min/week, which represents ~13.2 h/week of moderate-intensity or ~6.6 h/week of vigorous-intensity physical activity [although this large time difference probably results from over-reported MVPA (16, 17)]. The sex differences might be explained by the larger proportion of women self-reporting no MVPA. But notably, the difference persisted when including only participants with some MVPA, and when including only active participants. These data suggest that the sex gap in MVPA is so marked, that it even exists among active participants. Given the benefits of MVPA on several health outcomes (2–4), understanding the factors that explain the difference seems relevant. One possibility that has been raised is that women do less MVPA in the leisure domain (7). To properly test this hypothesis, herein we have analyzed absolute values of MVPA by domain, and also the relative contribution to total MVPA by domain. We discuss those approaches in the following two paragraphs.

Regarding the absolute values of MVPA, women had lower MVPA than men in all domains. This observation suggests that the sex gap is not domain-specific. MVPA was between 1.5 and 2.2-fold higher in men than women. The mean difference between sexes in MVPA was 2,236 MET × min/week in work/household, 568 MET × min/week in transport, and 352 MET × min/week in leisure domains. Similar patterns were observed herein when including only participants with some MVPA, and also in a previous study that included participants from 104 countries (10). Together, evidence indicates that the work/household domain accounts for most of the sex gap in MVPA. We found a similar prevalence of participants being “working, studying or searching for work” between men and women. Thus, differences in the job performed (probably due to cultural stereotypes) should explain the difference in MVPA at work/household. Noteworthy, the sex gap in MVPA was reduced among ≥65-year-old participants. This may relate to a higher prevalence of retired workers, thus supporting the relevance of the job performed for explaining the sex gap in MVPA. Future studies should test this idea.

Regarding the relative contribution to total MVPA by domain, we found that women had higher relative contribution by the transport domain, and lower by the leisure domain. These results agree with the observations of studies in other countries (9, 10). Note, however, that since MVPA was consistently lower in women than men, the relative contributions by domain cannot account for the sex gap in MVPA. For example, women may have a larger relative contribution (in %) in one specific domain, yet still have lower MVPA than men in that domain (in MET × min/week). Indeed, although the relative contribution by the transport domain was higher in women than men, MVPA in this domain was still lower in women. Another aspect worth considering when analyzing relative contributions is that participants with no MVPA must be excluded. Those participants have no MVPA to be distributed between domains. This reduces the sample size, especially in women, because the prevalence of no MVPA was higher in women than in men (29 and 18%, respectively). Our findings highlight the relevance of comparing MVPA by domain as both absolute and relative values to better understand sex differences.

Our analyses and interpretations intend to help close the sex gap in MVPA in Chile and elsewhere. Since the largest mean difference in MVPA was at work/household, promoting MVPA in this domain appears attractive. Designing works that promote adequate levels of physical activity has been recently proposed (18, 19). Notably, a meta-analysis showed that a high level of occupational physical activity was associated with a higher risk of all-cause mortality in men (20). The phenomenon has been named the “physical activity paradox”. Yet, in the same meta-analysis, a high level of occupational physical activity trended toward less risk of all-cause mortality in women (20). Whether these effects result from sex differences in the response to physical activity, differences in the type of job, or analytical aspects remain to be determined (20, 21). Another factor to consider for helping close the sex gap is the feasibility of increasing MVPA at work. Physical activity at work often depends more on the job's characteristics than on the will of the workers. Consequently, increasing MVPA in the transport and leisure domains may be more feasible. Of note, active transport has been associated with lower cardiovascular risk in women (22), while leisure MVPA has been consistently associated with reduced risk of all-cause mortality (23). Therefore, the promotion of MVPA in transport and leisure domains is relevant for women, especially for those with no MVPA. This is a first step to close the sex gap in MVPA. Re-designing urban environments to ease active transportation, and increasing social opportunities for women to engage in leisure MVPA are required (24). Since lower leisure MVPA has been associated with lower education, ensuring access to education may be also relevant (25, 26). Indeed, women had less education than men in our current analyses. Whatever the chosen domain to close the sex gap, strategies should mainly focus on the women who report no MVPA. Evidence suggests that the largest reduction in the risk of all-cause mortality occurs when transitioning from no MVPA to some MVPA (27). This supports that some physical activity is better than none (2).

One limitation of our study was that MVPA was self-reported. Self-reports are prone to memory and desirability biases, leading to over-reported MVPA (16, 17). Moreover, the GPAQ requires self-reporting activities that occurred in at least 10-min bouts. Whether these features partially explain the sex gap in MVPA is unknown. Yet that seems unlikely, because the sex gap has also been shown in studies that measured MVPA using accelerometers (28–30). Another limitation is that the GPAQ only collects information about MVPA. Studies using accelerometers demonstrate that light-intensity activity is associated with reduced risk—whereas sedentary behavior with increased risk—of all-cause mortality (27). To fully appreciate whether a sex gap in “overall daily activity” exists, 24-h measurements including all physical behaviors (sleep, sedentary behavior, and physical activity) are required (31). Indeed, using 24-h accelerometry measurements, women from the UK Biobank study have shown higher overall daily activity than men (32). Despite its limitations, the GPAQ seems appropriate to study MVPA by domains, because participants self-report their MVPA at work/household, for transport, and for leisure. This distinction cannot be achieved with accelerometers. Finally, note that our findings only apply to the Chilean context. Whether similar patterns would be observed in other countries is unknown.

In conclusion, by analyzing MVPA by domain along with the relative contribution to total MVPA by domain, we have dissected the sex gap in MVPA in the adults of Chile. We have shown that all physical activity domains account for the sex gap in MVPA. Closing the sex gap in MVPA would help reduce the global prevalence of physical inactivity, one of the WHO goals (8, 33). Our results suggest that public health strategies should promote MVPA in women in the work/household, transport, and leisure domains. Breaking cultural stereotypes, increasing opportunities for leisure, re-designing urban environments, and ensuring access to education can help that purpose.

## Data availability statement

Publicly available datasets were analyzed in this study. This data can be found here: http://epi.minsal.cl/encuestas-poblacionales/.

## Author contributions

MS-R: conceived the study, analyzed the data, interpreted the data, and drafted the manuscript. RF-V: conceived the study, analyzed the data, interpreted the data, and drafted the manuscript. All authors revised critically the manuscript, and approved the final version.

## Funding

ANID/CONICYT Iniciación 11180361 to RF-V.

## Conflict of interest

The authors declare that the research was conducted in the absence of any commercial or financial relationships that could be construed as a potential conflict of interest.

## Publisher's note

All claims expressed in this article are solely those of the authors and do not necessarily represent those of their affiliated organizations, or those of the publisher, the editors and the reviewers. Any product that may be evaluated in this article, or claim that may be made by its manufacturer, is not guaranteed or endorsed by the publisher.
