# Supplementary material for: Work/household, transport, and leisure domains account for the sex gap in physical activity in Chile
Source: Front Public Health. 2022 Sep 28;10:1011790. doi: 10.3389/fpubh.2022.1011790 (PMC9554212; doi:10.3389/fpubh.2022.1011790)
Supplement: Supplementary file 1 [file Data_Sheet_1.pdf]

Supplementary Table 1. STROBE checklist for reports of cross-sectional studies.

	Item No	Recommendation	Section
**Title and abstract**	1	(*a*) Indicate the study's design with a commonly used term in the title or the abstract	Abstract
		(*b*) Provide in the abstract an informative and balanced summary of what was done and what was found	Abstract
**Introduction**			
Background/rationale	2	Explain the scientific background and rationale for the investigation being reported	Introduction, 1st to 3rd paragraph
Objectives	3	State specific objectives, including any prespecified hypotheses	Introduction, 4th paragraph
**Methods**			
Study design	4	Present key elements of study design early in the paper	Methods, sub-section "Design and setting"
Setting	5	Describe the setting, locations, and relevant dates, including periods of recruitment, exposure, follow-up, and data collection	
Participants	6	(*a*) Give the eligibility criteria, and the sources and methods of selection of participants	Methods, sub-section "Participants"
Variables	7	Clearly define all outcomes, exposures, predictors, potential confounders, and effect modifiers. Give diagnostic criteria, if applicable	Methods, sub-sections "Sociodemographic data" and "MVPA by domain"
Data sources/ measurement	8*	For each variable of interest, give sources of data and details of methods of assessment (measurement). Describe comparability of assessment methods if there is more than one group	
Bias	9	Describe any efforts to address potential sources of bias	-
Study size	10	Explain how the study size was arrived at	Methods, sub-section "Design and setting"
Quantitative variables	11	Explain how quantitative variables were handled in the analyses. If applicable, describe which groupings were chosen and why	Methods, sub-section "Statistics"
Statistical methods	12	(*a*) Describe all statistical methods, including those used to control for confounding	
		(*b*) Describe any methods used to examine subgroups and interactions	
		(*c*) Explain how missing data were addressed	Methods, sub-section "Sociodemographic data"
		(*d*) If applicable, describe analytical methods taking account of sampling strategy	Methods, sub-section "Design and setting"
		(*e*) Describe any sensitivity analyses	-
**Results**			
Participants	13*	(a) Report numbers of individuals at each stage of study—eg numbers potentially eligible, examined for eligibility, confirmed eligible, included in the study, completing follow-up, and analysed	Results, sub-section "General characteristics of participants"
		(b) Give reasons for non-participation at each stage	
		(c) Consider use of a flow diagram	-

Descriptive data	14*	(a) Give characteristics of study participants (eg demographic, clinical, social) and information on exposures and potential confounders	Results, sub-section "General characteristics of participants" and Table 1
		(b) Indicate number of participants with missing data for each variable of interest	
Outcome data	15*	Report numbers of outcome events or summary measures	Results and Figures 1-3
Main results	16	(*a*) Give unadjusted estimates and, if applicable, confounder-adjusted estimates and their precision (eg, 95% confidence interval). Make clear which confounders were adjusted for and why they were included	Results, sub-section "MVPA by domain including all selected participants" and Figure 1
		(*b*) Report category boundaries when continuous variables were categorized	Methods, sub-sections "Sociodemographic data" and "MVPA by domain"
		(*c*) If relevant, consider translating estimates of relative risk into absolute risk for a meaningful time period	-
Other analyses	17	Report other analyses done—eg analyses of subgroups and interactions, and sensitivity analyses	Results, sub-sections "MVPA by domain including only participants with some MVPA" and "MVPA by domain including only participants categorized as active", and Supplementary Tables 2-4
**Discussion**			
Key results	18	Summarise key results with reference to study objectives	Discussion, 1st paragraph
Limitations	19	Discuss limitations of the study, taking into account sources of potential bias or imprecision. Discuss both direction and magnitude of any potential bias	Discussion, 6th paragraph
Interpretation	20	Give a cautious overall interpretation of results considering objectives, limitations, multiplicity of analyses, results from similar studies, and other relevant evidence	Discussion, 7th paragraph
Generalisability	21	Discuss the generalisability (external validity) of the study results	Discussion, 7th paragraph
**Other information**			
Funding	22	Give the source of funding and the role of the funders for the present study and, if applicable, for the original study on which the present article is based	Funding

Supplementary Table 2. Comparison of moderate-vigorous physical activity (MVPA) levels between all selected men (n = 1,812) and women (n = 3,244), stratified by age.

MVPA	Men				Women				*P*-value
	Mean or frequency	Median	25th percentile	75th percentile	Mean or frequency	Median	25th percentile	75th percentile	
18 to <25 years									
n	204	-	-	-	289	-	-	-	-
All domains, MET×min/week	7,239	3,360	1,090	9,600	3,424	1,200	170	4,320	<0.001
At work/household, MET×min/week	3,612	0	0	2,760	1,884	0	0	480	0.019
For transport, MET×min/week	2,073	960	300	2,400	1,047	480	0	1,200	<0.001
For leisure, MET×min/week	1,554	480	0	2,160	493	0	0	480	<0.001
Categories									
No MVPA, %	8.3	-	-	-	23.2	-	-	-	<0.001
Insufficiently active, %	6.9	-	-	-	14.5	-	-	-	-
Active, %	84.8	-	-	-	62.3	-	-	-	-
25 to <45 years									
n	527	-	-	-	974	-	-	-	-
All domains, MET×min/week	8,037	3,660	840	11,520	4,442	1,270	144	5,610	<0.001
At work/household, MET×min/week	5,113	0	0	7,200	2,633	0	0	1,920	<0.001
For transport, MET×min/week	1,918	600	0	1,680	1,325	420	0	1,200	0.002
For leisure, MET×min/week	1,007	0	0	1,440	484	0	0	0	<0.001
Categories									
No MVPA, %	13.5	-	-	-	24.3	-	-	-	<0.001
Insufficiently active, %	7.2	-	-	-	12.1	-	-	-	-
Active, %	79.3	-	-	-	63.6	-	-	-	-
45 to <65 years									
n	618	-	-	-	1,151	-	-	-	-
All domains, MET×min/week	8,256	3,360	480	12,650	4,232	1,200	0	5,760	<0.001
At work/household, MET×min/week	5,989	0	0	9,600	2,648	0	0	1,952	<0.001
For transport, MET×min/week	1,930	600	0	1,920	1,377	360	0	1,440	<0.001
For leisure, MET×min/week	337	0	0	0	207	0	0	0	<0.001
Categories									
No MVPA, %	18.1	-	-	-	27.3	-	-	-	<0.001
Insufficiently active, %	7.8	-	-	-	11.9	-	-	-	-
Active, %	74.1	-	-	-	60.8	-	-	-	-
≥65 years									
n	463	-	-	-	830	-	-	-	-
All domains, MET×min/week	3,467	840	0	3,600	2,178	480	0	2,214	<0.001
At work/household, MET×min/week	1,831	0	0	0	1,054	0	0	0	0.263
For transport, MET×min/week	1,355	560	0	1,680	967	200	0	960	<0.001
For leisure, MET×min/week	282	0	0	0	157	0	0	0	0.083
Categories									
No MVPA, %	25.5	-	-	-	37.2	-	-	-	<0.001
Insufficiently active, %	14.3	-	-	-	16.4	-	-	-	-
Active, %	60.3	-	-	-	46.4	-	-	-	-

Supplementary Table 3. Comparison of moderate-vigorous physical activity (MVPA) levels between men (n = 1,494) and women (n = 2,317) that reported some MVPA, stratified by age.

MVPA	Men				Women				*P*-value
	Mean or frequency	Median	25th percentile	75th percentile	Mean or frequency	Median	25th percentile	75th percentile	
18 to <25 years									
n	187	-	-	-	222	-	-	-	-
All domains, MET×min/week	7,897	4,780	1,680	10,560	4,458	2,000	840	5,800	<0.001
At work/household, MET×min/week	3,941	0	0	3,360	2,453	0	0	2,400	0.237
For transport, MET×min/week	2,261	1,200	420	2,880	1,363	600	300	1,680	<0.001
For leisure, MET×min/week	1,695	720	0	2,400	642	0	0	720	<0.001
At work/household, %	23.9	0.0	0.0	48.6	25.9	0.0	0.0	60.4	0.955
For transport, %	46.2	35.7	10.9	100.0	55.5	53.8	12.3	100.0	0.038
For leisure, %	29.9	17.5	0.0	55.9	18.6	0.0	0.0	30.0	<0.001
25 to <45 years									
n	456	-	-	-	737	-	-	-	-
All domains, MET×min/week	9,289	5,400	1,643	13,675	5,870	2,800	840	7,780	<0.001
At work/household, MET×min/week	5,909	330	0	9,600	3,480	0	0	3,840	<0.001
For transport, MET×min/week	2,216	840	0	2,495	1,751	720	280	1,680	0.265
For leisure, MET×min/week	1,163	310	0	1,440	640	0	0	480	<0.001
At work/household, %	38.8	16.2	0.0	83.7	31.0	0.0	0.0	74.8	0.001
For transport, %	37.1	20.0	0.0	76.7	54.5	51.2	7.9	100.0	<0.001
For leisure, %	24.1	3.2	0.0	39.2	14.5	0.0	0.0	12.5	<0.001
45 to <65 years									
n	506	-	-	-	837	-	-	-	-
All domains, MET×min/week	10,084	5,880	1,680	15,125	5,820	2,880	840	8,400	<0.001
At work/household, MET×min/week	7,315	1,860	0	12,360	3,641	0	0	5,040	<0.001
For transport, MET×min/week	2,357	840	240	3,360	1,893	812	280	1,680	0.091
For leisure, MET×min/week	412	0	0	240	285	0	0	0	<0.001
At work/household, %	46.1	50.0	0.0	92.8	36.4	0.0	0.0	85.6	<0.001
For transport, %	44.0	30.4	3.0	100.0	56.2	63.6	7.7	100.0	<0.001
For leisure, %	10.0	0.0	0.0	2.3	7.4	0.0	0.0	0.0	<0.001
≥65 years									
n	345	-	-	-	521	-	-	-	-
All domains, MET×min/week	4,653	1,680	720	5,760	3,470	1,680	560	4,200	0.044
At work/household, MET×min/week	2,457	0	0	1,560	1,680	0	0	860	0.798
For transport, MET×min/week	1,818	840	410	1,920	1,540	700	240	1,680	0.010
For leisure, MET×min/week	378	0	0	0	250	0	0	0	0.498
At work/household, %	23.7	0.0	0.0	50.0	25.7	0.0	0.0	50.9	0.284
For transport, %	68.3	100.0	28.3	100.0	66.5	100.0	25.0	100.0	0.613
For leisure, %	8.0	0.0	0.0	0.0	7.8	0.0	0.0	0.0	0.623

Supplementary Table 4. Comparison of moderate-vigorous physical activity (MVPA) levels between men (n = 1,328) and women (n = 1,884) categorized as active, stratified by age.

MVPA	Men				Women				*P*-value
	Mean or frequency	Median	25th percentile	75th percentile	Mean or frequency	Median	25th percentile	75th percentile	
18 to <25 years									
n	173	-	-	-	180	-	-	-	-
All domains, MET×min/week	8,505	5,496	1,920	11,490	5,414	3,240	1,440	7,290	0.001
At work/household, MET×min/week	4,257	0	0	4,560	3,022	0	0	3,780	0.781
For transport, MET×min/week	2,421	1,200	600	3,360	1,604	840	485	1,680	0.007
For leisure, MET×min/week	1,827	960	0	2,760	788	0	0	960	<0.001
At work/household, %	25.2	0.0	0.0	53.5	31.4	0.0	0.0	73.7	0.285
For transport, %	43.6	33.3	10.0	90.0	46.9	36.3	7.2	100.0	0.63
For leisure, %	31.2	22.2	0.0	56.3	21.7	0.0	0.0	40.1	0.001
25 to <45 years									
n	418	-	-	-	619	-	-	-	-
All domains, MET×min/week	10,102	6,420	2,160	15,120	6,921	3,720	1,620	9,120	<0.001
At work/household, MET×min/week	6,440	1,320	0	10,080	4,141	0	0	5,760	0.001
For transport, MET×min/week	2,402	996	150	3,000	2,026	840	400	1,680	0.400
For leisure, MET×min/week	1,260	480	0	1,680	755	0	0	720	<0.001
At work/household, %	40.6	26.7	0.0	84.0	36.3	0.0	0.0	81.1	0.084
For transport, %	35.8	20.0	1.0	69.8	48.3	34.1	5.5	100.0	<0.001
For leisure, %	23.6	4.4	0.0	37.9	15.4	0.0	0.0	17.5	<0.001
45 to <65 years									
n	458	-	-	-	700	-	-	-	-
All domains, MET×min/week	11,105	7,200	2,400	16,800	6,894	4,020	1,680	9,354	<0.001
At work/household, MET×min/week	8,077	3,360	0	13,440	4,349	720	0	6,000	<0.001
For transport, MET×min/week	2,577	1,160	360	3,360	2,208	960	400	2,520	0.347
For leisure, MET×min/week	451	0	0	330	337	0	0	0	0.001
At work/household, %	49.7	60.0	0.0	94.3	42.0	33.3	0.0	90.2	0.007
For transport, %	40.8	25.0	3.0	98.7	50.3	41.2	5.6	100.0	<0.001
For leisure, %	9.5	0.0	0.0	3.8	7.6	0.0	0.0	0.0	0.005
≥65 years									
n	279	-	-	-	385	-	-	-	-
All domains, MET×min/week	5,674	2,400	1,020	7,220	4,587	2,520	1,320	5,620	0.858
At work/household, MET×min/week	3,031	0	0	3,360	2,261	0	0	2,040	0.767
For transport, MET×min/week	2,175	1,200	720	2,520	1,995	1,120	560	2,400	0.233
For leisure, MET×min/week	468	0	0	0	330	0	0	0	0.479
At work/household, %	27.2	0.0	0.0	63.2	30.8	0.0	0.0	73.8	0.208
For transport, %	62.9	77.8	23.8	100.0	60.7	71.4	17.0	100.0	0.646
For leisure, %	9.9	0.0	0.0	0.0	8.6	0.0	0.0	0.0	0.486

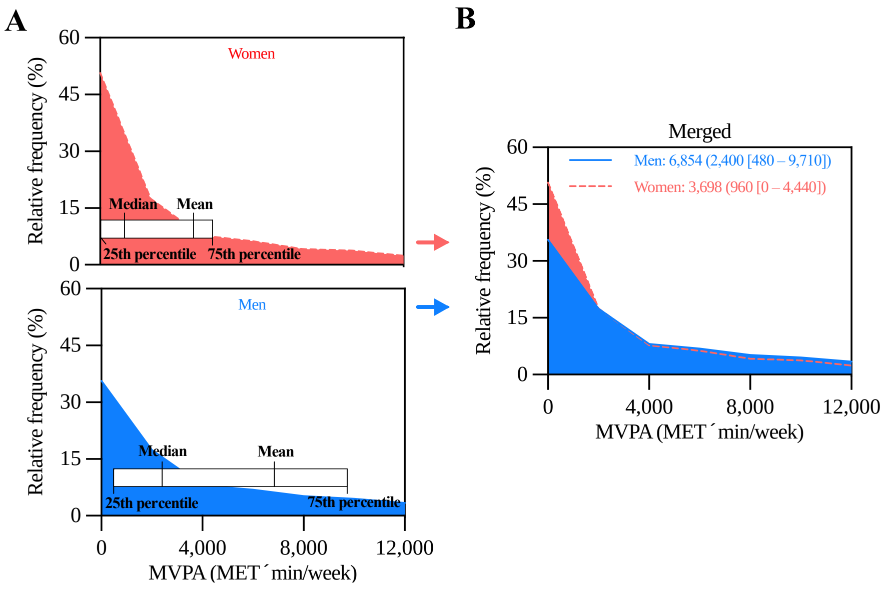

Supplementary Figure 1. Comparing highly skewed data using histograms of relative frequencies. (A) The histograms show the relative frequency distribution of participants (in %; *y*-axis) as a function of the level of moderate-vigorous physical activity (MVPA, in MET×min/week; *x*-axis). The more skewed the histogram to the left, the lower the MVPA of participants. This is because such a pattern indicates that a larger proportion of participants has low MVPA values. The mean, median, 25th percentile, and 75th percentile shown in the figure help summarize the pattern. In the example, data for women are more skewed to the left than data for men. Thus, there is a larger proportion of women with low MVPA values compared to men. This difference manifests in lower mean, median, 25th percentile, and 75th percentile in women than in men. (B) To compare the data between men and women in the manuscript, we merged the histograms, and presented the data as: mean (median [25th percentile – 75th percentile].

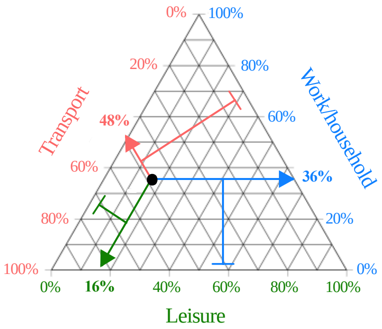

Supplementary Figure 2. Reading ternary plots of the relative contribution to total moderate-vigorous physical activity (MVPA) by domains. Ternary plots summarize the values of three variables whose sum is constant. In the context of physical activity, MVPA can be conducted at work/household, for transport, and for leisure. The sum of the MVPA in these three domains results in the total MVPA. Thus, each domain contributes a certain proportion (%) to the total MVPA (the 100%). The plot shows how to read the data for a group represented by the circle. For MVPA at work/household, with an arrow running parallel to the *x*-axis. For MVPA for transport, with an arrow running parallel to the right-hand axis. And for MVPA for leisure, with an arrow running parallel to the left-hand axis. In the example, the relative contribution to total MVPA is 36% for work/household, 48% for transport, and 16% for leisure domains.
